# Introduction of standardised packaging and availability of illicit cigarettes: a difference-in-difference analysis of European Union survey data 2015–2018

**DOI:** 10.1136/thoraxjnl-2020-215708

**Published:** 2020-10-22

**Authors:** Anthony A Laverty, Christopher Millett, Nicholas S Hopkinson, Filippos T Filippidis

**Affiliations:** 1 Public Health Policy Evaluation Unit, School of Public Health, Imperial College London, London, UK; 2 National Heart and Lung Institute, Imperial College London, London, UK

**Keywords:** tobacco control

## Abstract

Standardised packaging of tobacco products is intended to reduce the appeal of smoking, but the tobacco industry claims this increases illicit trade. We examined the percentage of people reporting being offered illicit cigarettes before and after full implementation of standardised packaging in the UK, Ireland and France and compared this to other European Union countries. Reported ever illicit cigarette exposure fell from 19.8% to 18.1% between 2015 and 2018 in the three countries fully implementing the policy, and from 19.6% to 17.0% in control countries (p for difference=0.320). Standardised packaging does not appear to increase the availability of illicit cigarettes.

## Introduction

In 2012 Australia became the first country to require standardised (or plain) packaging for tobacco products. The tobacco industry argued that this is ineffective^1^ and will increase illicit tobacco trade, which refers to tobacco which is counterfeited, smuggled or has evaded due taxes.^2^ Illicit trade may be problematic as it blunts impacts of tobacco taxation, reduces potential revenue for governments and increases tobacco access for youth and the poor.^2^ While academic research and the Chantler review concluded there was no evidence of an increase in illicit trade in Australia after implementation, there is limited independent evidence on the issue.^3 4^ The recent World Trade Organization decision that standardised packaging is consistent with international trade law may encourage implementation in other countries, but also misinformation and lobbying by the tobacco industry.^5^ We assessed whether full implementation of standardised packaging in the UK, France and Ireland was associated with a change in the frequency of being offered illicit cigarettes.

## Methods

We analysed individual-level data from waves 84.4 (collected November/December 2015; n=27 672) and 90.4 (December 2018; n=27 636) of Special Eurobarometer Surveys.^6 7^ Participants were interviewed face to face in their own home. Standardised packaging has been in force for cigarettes and rolling tobacco sold in France from January 2017, in the UK from May 2017 and for all tobacco products manufactured after 30 September 2017 in Ireland.

The outcome was reporting being offered illicit cigarettes, assessed with: ‘Have you ever been offered black market cigarettes to buy or smoke?’ Responses were ‘No, never’; ‘Yes, rarely (<once a month)’; ‘Yes, occasionally (1–3 times per month)’; and ‘Yes, frequently (once per week or more)’.

Sociodemographic data included age (15–24, 25–39, 40–54, 55+ years); sex; residence type (rural, town/suburb or city); age at completion of education (0–15, 16–19, 20+ years, still studying); occupation (employed, unemployed); cigarette/rolling tobacco smoking status (non-smoker, current and former smoker); and difficulty paying bills during the last year (almost never/rarely, occasionally, most of the time).

Country-level data on gross domestic product (GDP) per capita came from Eurostat and the corruption perception index (CPI) data from Transparency International.^8^ Tobacco Control Scale (TCS) scores (https://www.tobaccocontrolscale.org/) captured national tobacco control policies. We excluded the price element of the score and used weighted average price (WAP) of cigarettes provided by the European Commission and adjusted these for inflation using the Harmonised Index of Consumer Prices (https://ec.europa.eu/eurostat). We employed a difference-in-difference approach using a two-level ordered (random intercept) regression model. This accounts for clustering of individuals’ responses (first level) within countries (second level).

In the difference-in-difference model, we included a binary exposure variable (countries that fully implemented standardised packaging vs countries that did not), year (2015 vs 2018) and an interaction term between these two (the difference-in-difference estimate). The model included CPI, GDP per capita, TCS score and WAP (country level); age, sex, residence type, education, occupation, smoking status and difficulty paying bills (individual level). The three countries which fully implemented standardised packaging (intervention) were compared with the other 25 European Union (EU) countries (control). We also ran ordered regression models in each country adjusting for these factors.

Results are presented as population-weighted mean and adjusted odds ratios (aOR) with 95% CI. Ordered regression uses all of the responses to the outcome question and gives odds for being offered illicit cigarettes more often. ORs above 1 represent an increased frequency of being offered, and vice versa. Sensitivity analyses were performed using data from smokers only, excluding countries with land borders with non-EEA countries (previously linked to illicit cigarette availability) and excluding Hungary (which had a phased implementation of the policy, ending 2022).^8^

## Results

**Figure 1:**
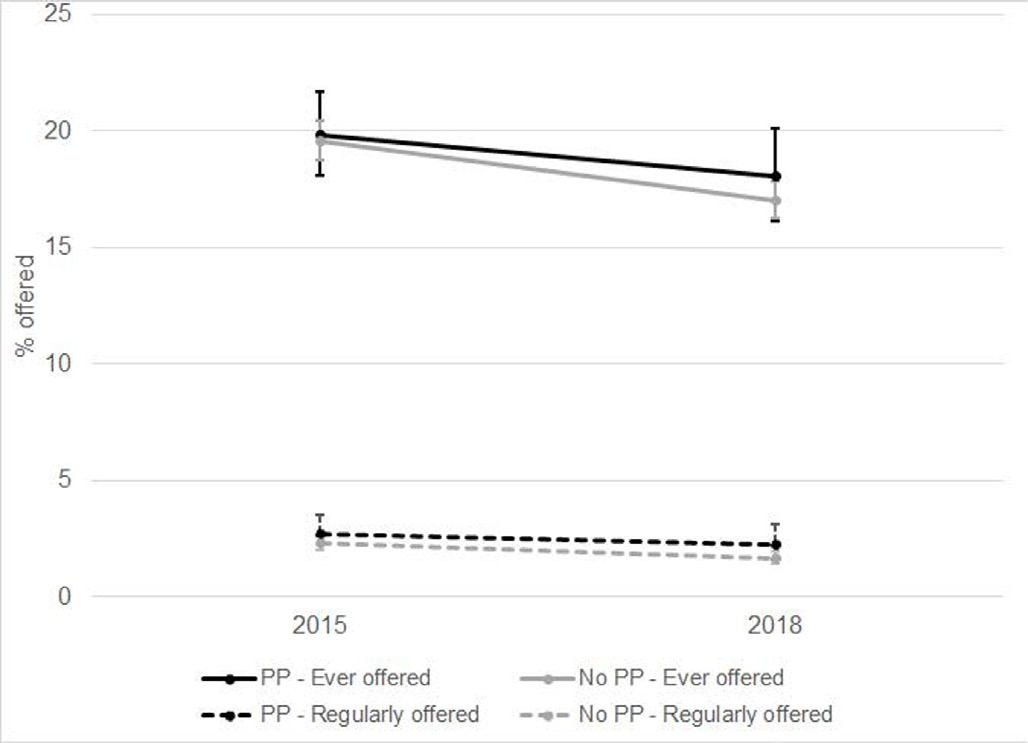
Weighted prevalence of having been offered illicit cigarettes in 2015 and 2018 in countries which introduced standardised packaging and control countries. PP, plain packaging.

In the three intervention countries 19.8% (95% CI 18.1 to20.4) of respondents were ever offered illicit cigarettes and 2.7% (95% CI 2.0% to 3.5%) regularly in 2015. In 2018, 18.1% (95% CI 16.2 to 20.1) of respondents were ever offered illicit cigarettes and 2.3% (95% CI 1.6% to 3.2%) regularly (figure 1).

In the 25 control countries, 19.6% (95% CI 18.7 to 20.4) of respondents were ever offered illicit cigarettes and 2.3% regularly (95% CI 2.0% to 2.6%) in 2015. In 2018, 17.0% (95% CI 16.2 to 17.8) of respondents were ever offered illicit cigarettes, 1.7% (95% CI 1.4% to 2.0%) regularly.

**Figure 2:**
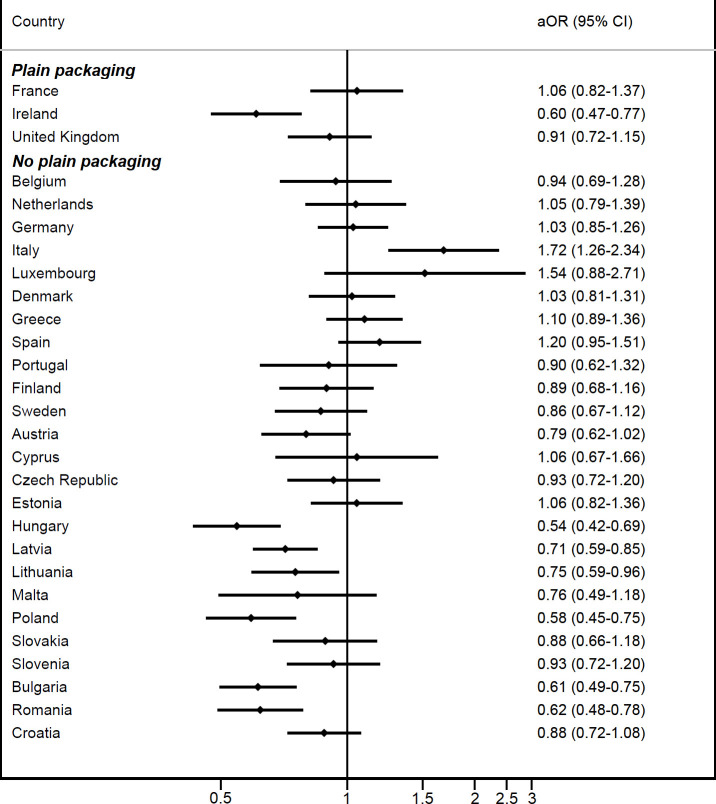
Changes in odds of having been offered illicit cigarettes more frequently between 2015 and 2018, by country. aOR, adjusted odds ratio.

**Figure 3:**
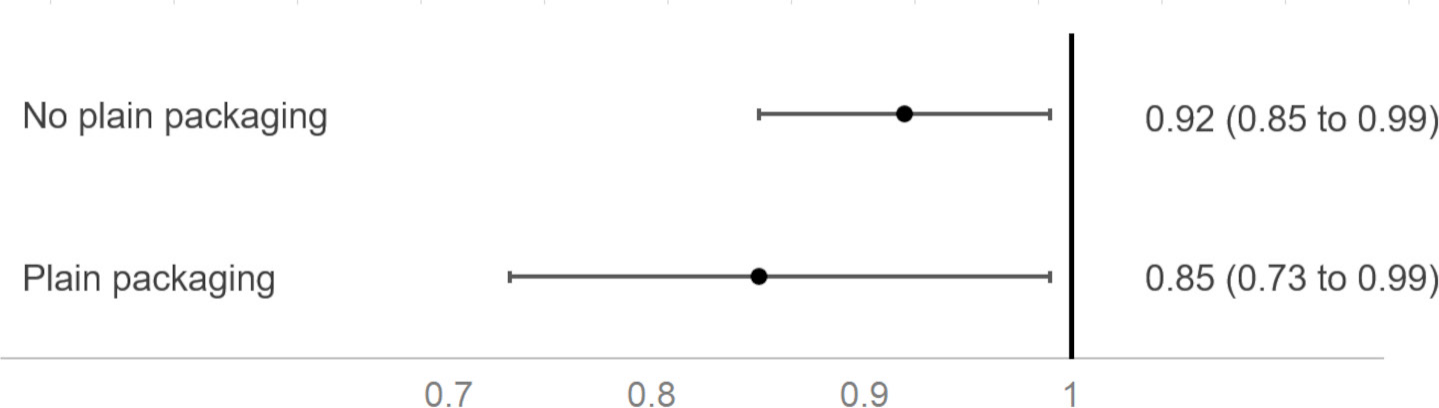
Changes in odds of having been offered illicit cigarettes more frequently between 2015 and 2018 in countries which introduced standardised packaging and those which did not.

Although changes from 2015 to 2018 varied across the EU (online supplemental table 1 and figure 2), the frequency of being offered illicit cigarettes fell between 2015 and 2018 in both control (aOR 0.92 (95% CI 0.85 to 0.99)) and intervention countries (aOR 0.85 (95% CI 0.73 to 0.99)). These two estimates were not statistically significantly different (aOR for interaction term: 0.93 (95% CI 0.80 to 1.07; p=0.320), figure 3).

10.1136/thoraxjnl-2020-215708.supp1 Supplementary data

Results for sensitivity analyses among smokers only, and excluding Hungary also found no differences between intervention and control countries (online supplemental tables 2 and 3). Analyses among countries with no non-EEA border suggested larger declines among countries implementing the policy (aOR for interaction 0.78 (95% CI 0.67 to 0.91), p=0.002).

## Discussion

This study is the first to assess whether levels of illicit trade in tobacco have risen after full implementation of standardised packaging in Europe. We found no evidence to suggest that this was the case.

This study used nationally representative data and a robust design with consistent outcome measures over time and between countries. Nonetheless, we relied on data from only two time points, meaning we could not assess trends in illicit tobacco before 2015. Rather than the more accepted term ‘illicit cigarettes’ the survey referred to ‘black market’ cigarettes, with participants not provided examples of these. The meaning of this term may differ between countries, thus cross-country comparisons of absolute percentages may not fully reflect the availability of illicit cigarettes. However, we compared within-country changes in a 3-year period, hence such differences should have minimal effects on our findings. We were also unable to assess possible impacts of standardised packaging on different types of illicit tobacco (such as counterfeit tobacco), or on rolling tobacco. These could be examined in future research as well as assessing actual use of illicit tobacco in addition to being offered it. While our analyses are based on self-reported data, Eurobarometer uses a consistent design and provides unique data on illicit trade, a topic known to be difficult to evaluate.^9 10^

## Conclusions

These results suggest that standardised packaging does not lead to an increase in smokers' exposure to illicit tobacco. Governments should therefore not be discouraged from implementing the measure on the basis of arguments that it will.
