# Supplementary material for: Introduction of standardised packaging and availability of illicit cigarettes: a difference-in-difference analysis of European Union survey data 2015–2018
Source: Thorax. 2020 Oct 22;76(1):89–91. doi: 10.1136/thoraxjnl-2020-215708 (PMC7803897; doi:10.1136/thoraxjnl-2020-215708)
Supplement: Supplementary data [file thoraxjnl-2020-215708supp001.pdf]

Supplementary Table 1. Percentages of reporting being offered illicit cigarettes by individual country and year

	Offered rarely, occasionally or regularly Weighted % (95% CI)		Offered occasionally or regularly Weighted % (95% CI)		Offered regularly Weighted % (95% CI)	
	2015	2018	2015	2018	2015	2018
COUNTRIES IMPLEMENTING STANDARDISED PACKAGING						
France	18.3 (15.9-21.0)	19.4 (16.8-22.4)	4.1 (2.9-5.7)	5.5 (4.1-7.5)	1.5 (0.9-2.6)	1.6 (0.9-3.0)
Ireland	25.6 (22.8-28.5)	15.1 (12.8-17.7)	10.1 (8.3-12.3)	5.2 (3.8-7.0)	3.4 (2.4-4.8)	2.2 (1.3-3.5)
United Kingdom	21.0 (18.5-23.8)	16.8 (14.0-20.1)	9.9 (8.1-12.1)	7.6 (5.7-9.9)	3.9 (2.8-5.4)	2.9 (1.9-4.4)
COUNTRIES WITHOUT STANDARDISED PACKAGING						
Austria	23.6 (20.9-26.6)	18.1 (15.8-20.7)	5.5 (4.2-7.2)	4.8 (3.6-6.3)	1.3 (0.7-2.2)	0.5 (0.2-1.2)
Belgium	9.4 (7.7-11.5)	9.3 (7.6-11.4)	1.8 (1.1-2.8)	1.1 (0.7-1.8)	0.9 (0.5-1.6)	0.5 (0.2-1.2)
Bulgaria	34.2 (31.3-37.2)	27.1 (24.3-30.0)	18.8 (16.5-21.3)	10.3 (8.5-12.4)	8.8 (7.2-10.7)	2.8 (1.9-4.1)
Croatia	33.8 (30.8-37.0)	32.4 (29.3-35.5)	14.6 (12.4-17.0)	12.6 (10.6-14.9)	3.2 (2.3-4.6)	3.8 (2.8-5.1)
Cyprus	12.9 (9.9-16.5)	11.7 (8.7-15.5)	6.7 (4.5-9.8)	3.3 (1.9-5.5)	2.4 (1.2-4.9)	1.7 (0.8-3.6)
Czech Republic	18.4 (15.9-21.0)	15.9 (13.7-18.5)	4.8 (3.6-6.3)	4.6 (3.4-6.2)	1.7 (1.1-2.7)	1.3 (0.7-2.3)
Denmark	20.1 (17.5-23.0)	22.3 (19.2-25.8)	3.4 (2.4-5.0)	5.6 (4.1-7.8)	0.8 (0.4-1.6)	2.9 (1.8-4.5)
Estonia	21.8 (19.0-24.8)	22.0 (19.1-25.2)	5.3 (3.9-7.1)	6.4 (4.8-8.5)	2.4 (1.5-3.9)	2.2 (1.4-3.4)
Finland	19.2 (16.6-22.2)	15.5 (13.1-18.3)	3.1 (2.1-4.5)	2.6 (1.7-4.1)	0.6 (0.2-1.3)	1.1 (0.5-2.2)
Germany	19.4 (17.1-21.9)	17.6 (15.4-20.0)	4.3 (3.3-5.7)	4.5 (3.4-5.9)	1.5 (0.9-2.4)	1.3 (0.8-2.1)
Greece	27.6 (24.8-30.7)	28.9 (25.9-32.2)	9.6 (7.8-11.7)	9.4 (7.4-11.7)	3.6 (2.6-5.0)	2.3 (1.4-3.8)
Hungary*	29.1 (26.0-32.3)	16.7 (14.4-19.4)	9.3 (7.4-11.6)	4.0 (2.8-5.5)	1.9 (1.2-3.1)	0.7 (0.3-1.8)
Italy	10.3 (8.3-12.7)	12.4 (10.4-14.7)	3.1 (2.1-4.5)	3.9 (2.8-5.3)	0.5 (0.2-1.3)	0.6 (0.3-1.3)
Latvia	56.3 (53.0-59.7)	43.4 (40.1-46.8)	23.2 (20.3-26.2)	16.3 (14.0-18.9)	10.4 (8.4-12.7)	6.9 (5.3-8.8)
Lithuania	35.7 (32.2-39.3)	28.8 (25.7-32.1)	16.1 (13.5-19.1)	13.1 (10.9-15.7)	6.8 (5.1-9.0)	6.8 (5.2-8.9)
Luxembourg	5.9 (3.8-8.9)	8.3 (5.8-11.7)	0 (0-0)	1.4 (0.6-3.2)	0 (0-0)	0.5 (0.1-2.3)
Malta	15.8 (12.4-20.0)	11.6 (8.4-15.9)	5.2 (3.3-8.3)	5.9 (3.7-9.5)	1.4 (0.7-3.0)	1.0 (0.4-2.4)
Netherlands	14.7 (12.4-17.3)	11.2 (9.2-13.7)	3.2 (2.1-4.7)	1.8 (1.1-3.1)	0.9 (0.5-1.7)	0.8 (0.3-2.1)
Poland	23.8 (21.1-26.8)	16.3 (14.0-18.9)	10.2 (8.3-12.3)	5.6 (4.3-7.3)	3.9 (2.8-5.4)	2.5 (1.6-3.8)
Portugal	9.6 (7.9-11.7)	7.5 (5.9-9.5)	2.1 (1.3-3.3)	1.6 (0.9-2.7)	1.1 (0.6-2.0)	0.5 (0.2-1.3)
Romania	26.9 (24.1-30.0)	16.1 (14.0-18.6)	14.2 (12.0-16.6)	6.8 (5.4-8.5)	6.1 (4.8-7.9)	1.9 (1.2-3.0)
Slovakia	18.5 (16.0-21.4)	15.4 (12.9-18.3)	4.0 (2.8-5.6)	3.8 (2.6-5.5)	1.1 (0.6-2.2)	0.6 (0.3-1.4)
Slovenia	18.5 (16.0-21.3)	16.5 (14.2-19.0)	5.6 (4.2-7.4)	3.9 (2.8-5.3)	1.9 (1.2-2.9)	1.5 (0.8-2.5)
Spain	21.7 (19.0-24.8)	21.4 (18.9-24.1)	6.6 (5.0-8.6)	9.0 (7.4-11.0)	3.3 (2.3-4.8)	3.4 (2.5-4.7)
Sweden	17.2 (14.1-20.8)	15.4 (12.9-18.3)	2.1 (1.3-3.6)	2.6 (1.6-4.1)	0.9 (0.4-2.1)	0.8 (0.4-2.0)

* Hungary introduced legislation so that from August 2016 all new tobacco products had to be in standardised packaging. Initially, all tobacco products were to be in standardised packaging from May 2019, although this date has now been postponed until 1 January 2022.

Supplementary Table 2. Full regression results and sensitivity analyses from ordered logistic regression of changes in odds of frequency of having been offered illicit cigarettes between 2015 and 2018 in countries which introduced plain packaging and those which didn't.

		Entire sample (n=52,889) aOR (95% CI)	Current smokers only (n=13,665) aOR (95% CI)	Countries without land borders with non-EEA countries only (n=30,677) aOR (95% CI)
**Difference in difference estimate (Interaction term (Country with plain packaging*Year))**		0.93 (0.80-1.07)	1.10 (0.87-1.38)	0.78 (0.67-0.91)
p-value for interaction term		0.320	0.426	0.002
Country with plain packaging				
	No	ref	ref	ref
	Yes	1.25 (0.60-2.58)	1.20 (0.51-2.85)	1.74 (0.94-3.23)
Year				
	2015	ref	ref	ref
	2018	0.92 (0.85-0.99)	0.92 (0.82-1.03)	1.16 (1.05-1.30)
Smoking				
	Never smoker	ref	-	ref
	Former smoker	4.60 (4.31-4.91)	-	4.48 (4.10-4.90)
	Current smoker	7.36 (6.93-7.83)	-	6.27 (5.75-6.85)
Sex				
	Male	ref	ref	ref
	Female	0.58 (0.55-0.61)	0.56 (0.52-0.60)	0.54 (0.50-0.57)
Age group (in years)				
	15-24	ref	ref	ref
	25-39	1.15 (1.03-1.28)	1.03 (0.89-1.19)	1.12 (0.96-1.31)
	40-54	1.08 (0.97-1.22)	1.00 (0.87-1.17)	0.96 (0.82-1.13)
	55+	0.72 (0.64-0.81)	0.82 (0.70-0.95)	0.53 (0.45-0.62)
Area of residence				
	Rural (ref)	ref	ref	ref
	Small city	1.21 (1.14-1.28)	1.23 (1.12-1.34)	1.19 (1.09-1.29)
	Urban	1.42 (1.34-1.51)	1.36 (1.24-1.49)	1.46 (1.33-1.59)
Difficulty paying bills				
	Never/Almost never	ref	ref	ref
	From time to time	1.31 (1.24-1.38)	1.43 (1.32-1.56)	1.30 (1.19-1.41)
	Most of the time	1.88 (1.74-2.04)	2.24 (2.01-2.50)	1.97 (1.74-2.21)
Age when stopped full-time education (in years)				
	≤15	ref	ref	ref
	16-19	1.23 (1.14-1.33)	1.08 (0.96-1.20)	1.17 (1.05-1.29)
	≥20	1.18 (1.09-1.28)	1.02 (0.90-1.15)	1.03 (0.93-1.15)
	Still studying	1.24 (1.06-1.44)	0.90 (0.72-1.12)	1.10 (0.90-1.34)
Employment				
	Employed	ref	ref	ref
	Unemployed	1.26 (1.15-1.37)	1.22 (1.09-1.36)	1.23 (1.09-1.40)
Weighted Average Price (per 1 EUR)		1.00 (0.91-1.09)	1.05 (0.92-1.20)	1.03 (0.94-1.12)
Gross Domestic Product (per 1,000 EUR)		0.98 (0.97-0.99)	0.97 (0.96-0.99)	0.98 (0.97-0.99)
Corruption Perception Index (per 10 points)		1.16 (1.02-1.32)	1.13 (0.96-1.33)	1.33 (1.14-1.56)
Tobacco Control Scale score		1.01 (0.99-1.02)	1.00 (0.99-1.02)	0.99 (0.97-1.00)

aOR = adjusted odds ratio

Result from ordered logistic regression adjusted for factors at the individual and country level.

Ordered logistic regression estimates represent odds of being in higher category of having been offered illicit cigarettes using responses never; rarely; occasionally; frequently. Estimates greater than one representing greater frequency of being offered illicit cigarettes and vice versa

Individual level factors: age, sex, residence type (rural, small city, urban), age when completed education*, employment (yes, no), smoking status (current, former, never), difficulty paying bills (never/almost never, from time to time, most of the time).*

Country level factors: Corruption Perception Index, GDP per capita, tobacco control score (excluding price) and weighted average price of cigarettes)

Supplementary Table 3. Difference-in-Difference regression results excluding Hungary* from ordered logistic regression of changes in odds of frequency of having been offered illicit cigarettes between 2015 and 2018 in countries which introduced plain packaging and those which didn't.

		Sample = 50814<br>aOR (95% CI)
Difference in difference estimate (Interaction term (Country with plain packaging*Year))		0.91 (0.79-1.06)
p-value for interaction term		0.236
Country with plain packaging		
	No	Ref
	Yes	1.31 (0.63-1.71)
Year		
	2015	Ref
	2018	0.92 (0.85-0.99)

* Hungary introduced legislation so that from August 2016 all new tobacco products had to be in standardised packaging. Initially, all tobacco products were to be in standardised packaging from May2019, although this date has now been postponed until 1 January 2022.
